# Insights into Hospitalized Children with Urinary Tract Infections: Epidemiology and Antimicrobial Resistance Patterns in Israel—A Single Center Study

**DOI:** 10.3390/children11091142

**Published:** 2024-09-20

**Authors:** Hussein Zaitoon, Jenny Garkaby, Basheer Nassrallah, Livnat Sharkansky, Morya Shnaider, Irina Chistyakov, Jacob Genizi, Keren Nathan

**Affiliations:** 1 Department of Pediatrics, Bnai Zion Medical Center, Haifa 3104802, Israel; husseinz@tlvmc.gov.il (H.Z.); garkabyj@mcmaster.ca (J.G.); basheer.nassrallah@b-zion.org.il (B.N.); livnat.sharkansky@b-zion.org.il (L.S.); morya.shnaider@b-zion.org.il (M.S.); ira.chist@b-zion.org.il (I.C.); keren.nathan@b-zion.org.il (K.N.); 2 The Ruth and Bruce Rappaport Faculty of Medicine, Technion-Israel Institute of Technology, Haifa 3109601, Israel

**Keywords:** urinary tract infection, epidemiology, antimicrobial susceptibility, antimicrobial resistance

## Abstract

**Background:** The escalating resistance of uropathogens in pediatric febrile urinary tract infection (F-UTI) is a global concern. This study examined changing trends in F-UTI epidemiology and resistance patterns among Israeli pediatric inpatients over a decade. **Methods:** Demographic, clinical, and laboratory data for children between 3 months and 18 years old with febrile UTI from 2010 to 2021 were retrieved from electronic medical records. **Results:** A total of 761 cases of F-UTI were identified (702 females, mean age 43 months). *Escherichia coli* was the most common pathogen (85.9%), followed by *Pseudomonas aeruginosa* (3.5%) and *Klebsiella pneumoniae* (3.4%). Compared with the non-complicated UTI group, the complicated UTI group had significantly higher rates of *Pseudomonas aeruginosa* (5.3% vs. 1.0%, *p* = 0.002) and *Klebsiella pneumoniae* (4.6% vs. 1.6%, *p* = 0.03). Antibiotic resistance analysis revealed significant differences between the groups: resistance to cephalexin was higher in the complicated UTI group (19.3%) compared with the non-complicated UTI group (13.4%, *p* = 0.03). Notably, relatively low resistance rates were observed for ceftriaxone (4.4%) and gentamicin (6.0%). Over time, a significant decreasing trend in resistance to ampicillin was observed (slope = −0.0193, *p* = 0.011). No significant trends were found for trimethoprim–sulfamethoxazole, cephalexin, amoxicillin–clavulanic acid, ceftriaxone, and cefuroxime. **Conclusions:** Significant differences in pathogen distribution and resistance patterns between complicated UTI and non-complicated UTI groups highlight the need for continuous resistance monitoring and adherence to local guidelines. For the treatment of severe community F-UTI, ceftriaxone could be a reasonable option for first-onset F-UTI. Further studies are needed to implement antibiotic stewardship and optimize usage.

## 1. Introduction

Febrile urinary tract infection (F-UTI) is a prevalent bacterial infection in children, particularly during the first few months of life, and it represents a serious health concern [1]. Although F-UTI is highly prevalent among infants, the diagnosis in preverbal age groups remains challenging due to non-specific clinical features such as fever without a focus [2,3,4]. Early diagnosis and treatment are essential for the prevention of renal damage and long-term scarring [5,6]. To ensure appropriate and effective treatment, knowledge of local uropathogens and susceptibility patterns are required.

Previous reports on F-UTI show concerning evidence regarding the increasing resistance patterns of uropathogens [7,8,9,10,11,12]. The most common resistance patterns are the beta-lactamase production mechanisms and the extended spectrum beta-lactamase (ESBL) mechanisms. Risk factors such as previous antibiotic use and socioeconomic status are associated with the presence of such resistance, which is found to be increasing in the pediatric population in Israel [13,14] and other places [15]. Some uropathogens present with combinations of several mechanisms, including multidrug resistance (MDR) and extensive drug resistance (XDR) [16]. The increasing uropathogen resistance challenges the choice of empirical treatment for F-UTI.

The increase in the utilization of antimicrobial stewardship programs resulted in a shift in antimicrobial choices. The current recommendations for community-acquired F-UTI treatment is either a first or second-generation cephalosporin, amoxicillin–clavulanic acid, or trimethoprim–sulfamethoxazole (TMP-SMX) [17]. For inpatient empirical treatment, ceftriaxone or aminoglycosides are recommended.

The Israeli population has the highest rates of circumcised boys. This procedure has been linked to a lower incidence of F-UTI [18].

This study examines the evolving epidemiology and resistance patterns of F-UTI in pediatric patients at a community hospital over the past decade. Our objectives are to evaluate changes in resistance patterns following the implementation of local treatment guidelines and to assess the appropriateness of empirical therapies used in treating F-UTI.

## 2. Materials and Methods

### 2.1. Patient Selection and Data Collection

In this retrospective, single-center observational study we reviewed the charts of patients admitted for F-UTI between 2010 and 2021. Inclusion criteria comprised children younger than 18 years old who presented with fever, abnormal urinalysis, and a positive urine culture. All children included in the study had suspected UTIs based on their urinalysis results, which prompted the collection of urine cultures. Demographic, clinical, and laboratory data, including uropathogen resistance patterns, were collected from the patients’ charts.

Urine culture techniques were chosen based on the child’s age. For children younger than 3 years old, urethral catheterization was primarily used. However, in a few cases involving infants younger than 6 months old, where the small size of the urethra posed challenges, the suprapubic aspiration technique was employed. Suprapubic aspiration is considered a last resort for obtaining urine samples due to its invasive nature. For older, toilet-trained children, urine samples were collected using the midstream clean-catch method. Urine cultures were inoculated on culture media and incubated for 24 h. F-UTI was defined as the presence of more than 10^3^ CFU/mL in a catheter sample and more than 10^5^ CFU/mL in a midstream clean-catch sample [17,19]. Polymicrobial and fungal cultures were excluded along with non-sufficient colony numbers to diagnose F-UTI.

Since we aimed to examine changes in resistance patterns and to assess the appropriateness of empirical therapies used in treating F-UTI, infants younger than 3 months old were excluded due to the different management protocols that reflect coverage for maternal birth canal pathogens rather than pathogens in the pediatric microbial community.

The minimum inhibitory concentration (MIC) cutoff for antimicrobial susceptibility was determined from the Clinical Laboratory Standards Institute (CLSI) guidelines [20], which are updated every year and implemented in the VITEK 2 system, as mentioned below. According to these guidelines, bacteria were classified as susceptible, intermediate, or resistant based on the CLSI breakpoints. For the purposes of this analysis, both intermediate bacteria with MIC close to the resistance breakpoint and bacteria with resistant classifications were combined into a single group and considered resistant.

Bacteria were identified and their antimicrobial susceptibility including ESBL was tested using the automated VITEK 2 compact system (bioMérieux, Marcy-l’Étoile, France). This integrated modular system includes a filling–sealer unit, a reader–incubator, a computer control module, a data terminal, and a multicopy printer. It uses optical density-based technology to detect bacterial growth and metabolic changes. The Antimicrobial Susceptibility Test (ASR) cards that were used for Gram-negative bacteria were ASTN270 and ASTN395; for non-fermenters, ASTN 114 and ASTN 308 were used; and for Gram-positive bacteria, ASTP 584 and ASTP 649 were used. This study evaluated the effectiveness of various antimicrobial agents in treating F-UTI in hospitalized children. For Gram-negative bacteria, the antibiotics tested included meropenem, imipenem, amikacin, piperacillin–tazobactam, cefoperazone–sulbactam, ampicillin–sulbactam, nitrofurantoin, cefoxitin, tobramycin, ceftazidime, cefepime, gentamicin, ceftriaxone, cefuroxime, cefotaxime, sulfamethoxazole–trimethoprim, levofloxacin, ciprofloxacin, cefazolin, and ampicillin. For Gram-positive bacteria, the antibiotics tested included linezolid, vancomycin, teicoplanin, streptomycin, gentamicin, nitrofurantoin, rifampicin, moxifloxacin, levofloxacin, erythromycin, ampicillin, penicillin, ciprofloxacin, and clindamycin.

Clinical improvement was defined as fever resolution within the last 24 h and improvement in specific blood test inflammation markers such as C-reactive protein (CRP) levels (normal range 0–6 mg/L), which were typically retested 48–72 h after the initial test and before discharge. They were also tested if the patient’s condition worsened in order to monitor treatment effectiveness and to detect any deterioration.

We classified the study participants into two groups: the complicated UTI group, which included children with abnormal urinary tract anatomy, children with recurrent UTIs, and boys with UTIs; and the non-complicated UTI group, which included otherwise healthy individuals with normal urinary tract anatomy and no underlying risk factors. Recurrent F-UTI was defined as having at least one previous episode with a documented positive urine culture, characterized by significant bacterial growth (e.g., >10^5^ colony-forming units/mL), within the past 12 months.

Besides CRP, other blood analysis results collected at presentation to the emergency department were included, such as creatinine and urea levels as markers for kidney function; these tests were processed in a Cobas 6000 machine (Roche diagnostics, Basel, Switzerland). Creatinine and urea levels were primarily tested at presentation and before discharge using laboratory reference ranges that are age-dependent. For creatinine, the normal levels are 0.2–0.4 mg/dL for infants and 0.3–0.7 mg/dL for children aged 1–12 years. For urea, the normal levels are 3–15 mg/dL for infants and 5–20 mg/dL for older children.

### 2.2. Ethical Approval and Informed Consent

The study was approved by the institutional review board (Approval 0136-22-BNZ). Patient consent was waived due to the retrospective nature of the study.

### 2.3. Statistical Methods

Categorical data were presented as frequencies and percents. Continuous data were presented as medians with interquartile ranges. Differences in F-UTI etiology and resistance patterns between age groups were determined by the Fisher’s exact test. Comparison of demographic and clinical data between complicated and non-complicated UTI groups was performed using the Wilcoxon two-sample test for continuous data; the chi-square or Fisher’s exact test was used in the case of rare occurrences. The chi-square test for trend in proportions was used to investigate antibiotic resistance over the 11 years. A *p* value of <0.05 was considered to be statistically significant. All statistical analyses were performed using SPSS version 24 (IBM Corp, Armonk, NY, USA).

## 3. Results

### 3.1. Patient and Clinical Characteristics

**Figure 1:**
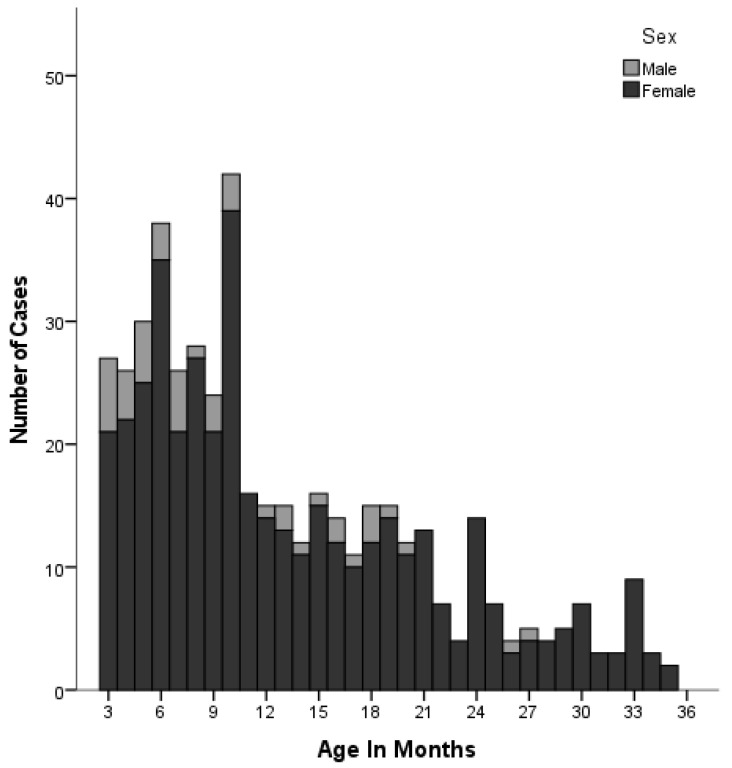
Age (months) and sex distribution of children with F-UTI.

There were 761 cases of F-UTI (702 females, mean age 43 months) between 2010 and 2021. Of these cases, 30.2% (n = 230) were aged 3–11 months, 25.2% (n = 192) were aged 1–2 years, and 17.9% (n = 136) were aged 3–4 years (Figure 1). The median age of male vs. female children diagnosed with F-UTI was significantly lower for males (12.5 vs. 20.0 months; Z = −2.636, *p* = 0.008). Circumcision was performed in 43 (72.8%) of the 59 males with F-UTI. Among the circumcised boys, 22 (51.1%) had a urinary tract anomaly. Of the 16 uncircumcised boys, 8 (50%) had a urinary tract anomaly. Overall, 14 boys (23.7%) experienced recurrent infections.

Of the 761 cases, 308 (40.5%) had a non-complicated UTI and 453 (59.5%) had a complicated UTI. The complicated UTI group included 59 boys. Among the 394 girls in the complicated UTI group, 177 had recurrent infections and 250 had urinary tract pathologies, with 33 girls having both urinary tract pathologies and recurrent infections. The urinary tract pathologies are presented in Table 1. Among these, 11 cases (2.5%) were identified as vesico-ureteric reflux, which were distributed as follows: 1 patient had grade 4 reflux, 3 patients had grade 3 reflux, and 7 patients had grade 2 reflux.

Nearly 7.7% of the complicated UTI cases (35/452) had their infection while taking prophylactic antibiotics. Recurrent F-UTI overall was diagnosed among 192 (25.2%) patients, with 32.8% of patients experiencing a recurrence with the same pathogen.

The median length of treatment during hospitalization for the whole cohort was 3 days (range 0–13 days).

### 3.2. Pathogens

The most common pathogen for all age groups was *Escherichia coli* (654; 85.9%). This was followed by *Pseudomonas aeruginosa* (27; 3.5%) and *Klebsiella pneumoniae* (26; 3.4%) (Table 1). The distribution of pathogens differed significantly between the complicated UTI group and the non-complicated UTI group: a higher prevalence of *Klebsiella pneumoniae* (χ^2^ = 5.04, *p* = 0.025) and *P. aeruginosa* (χ^2^ = 10.02, *p* = 0.002) was observed in the complicated UTI group compared with their non-complicated UTI counterparts, while *E. coli* was more prevalent in the non-complicated UTI group (χ^2^ = 20.49, *p* < 0.0001) (Table 1). Additionally, the complicated UTI group experienced longer treatment times during hospitalization (mean 3.2 days vs. mean 3.1 days, Z = 2.06, *p* = 0.04).

When examining the age distribution of uropathogens, the highest rate of *E. coli* was observed in children aged 1–2 years (Table 2). There was a statistically significant difference in the incidence of *Proteus mirabilis* (χ^2^ = 11.46, Fisher’s exact *p* = 0.033) and *Staphylococcus aureus* (χ^2^ = 10.78, Fisher’s exact *p* = 0.008); however, Bonferroni post-hoc testing did not indicate specific age differences for any pathogen.

### 3.3. Antibiotic Resistance

Inpatient parenteral empirical therapy was given to all children. Further, 436 children were treated with an aminoglycoside containing regimen (ACR) (57.2%) and 204 (26.8%) were treated with ceftriaxone. The remaining children were treated with combinations of other antimicrobials. Length of stay in the hospital was significantly longer for patients treated with ACR compared with those treated with ceftriaxone (3.1 vs. 3.7, Z = −6.42, *p* < 0.001), with higher pretreatment creatinine levels noted among children treated with ACR (Z = 2.82, *p* = 0.005).

Over the study period, *E. coli* resistance to gentamicin showed a diverse pattern between 1.4% and 10.3% resistance and ceftriaxone had 0.0% to 8.9% resistance. Gentamicin (N = 632), ceftriaxone (N = 597), ampicillin (N = 579), amoxicillin–clavulanic acid (N = 600), ciprofloxacin (N = 472), piperacillin–tazobactam (N = 445), meropenem (N = 443), TMP-SMX (N = 436), ceftazidime (N = 437), and ertapenem (N = 421) were the most common IV antimicrobial agents tested.

Only 22 urine culture results (2.9%) yielded Gram-positive uropathogens. Therefore, all subsequent resistance analysis results will focus exclusively on the antibiogram of Gram-negative uropathogens.

The analysis of antimicrobial resistance in pediatric patients with F-UTI revealed a significant difference in the resistance to cephalexin, with 19.3% resistance observed in complicated UTI patients compared with 13.4% in the non-complicated UTI patients (*p* = 0.03). Other antibiotics, including ampicillin, TMP-SMX, amoxicillin–clavulanic acid, cefuroxime, ciprofloxacin, nitrofurantoin, ceftriaxone, and gentamicin, showed no statistically significant differences in resistance between the complicated UTI and the non-complicated UTI groups, although overall resistance rates varied and several trends were indicated. Overall resistance from all Gram-negative bacterial cultures is presented in Table 3. The most commonly used empiric parenteral antibiotics, ceftriaxone (4.4%) and gentamicin (6%), had relatively low resistance rates (Table 3).

We compared the antibiotic resistance patterns of the non-complicated UTI group vs. the recurrent F-UTI group only. Resistance rates to TMP-SMX, cephalexin, and cefuroxime were found to be significantly higher in the recurrent F-UTI group (34.3% vs. 21.7%, χ^2^ = 6.85, *p* = 0.01; 20.0 vs. 8.8%, χ^2^ = 4.25, *p* = 0.039; 11.3 vs. 4.3%, χ^2^ = 4.78, *p* = 0.03). A smaller non-significant difference in antibiotic resistance was found among amoxicillin–clavulanic acid (10.7 vs. 6.3%, χ^2^ = 5.06, *p* = 0.08) and ceftriaxone (7.3% vs. 3.3%, χ^2^ = 4.61, *p* = 0.07). There were no significant differences in other antibiotics including gentamicin (6.0% vs. 5.3%, χ^2^ = 0.12, *p* = 0.73).

**Figure 2:**
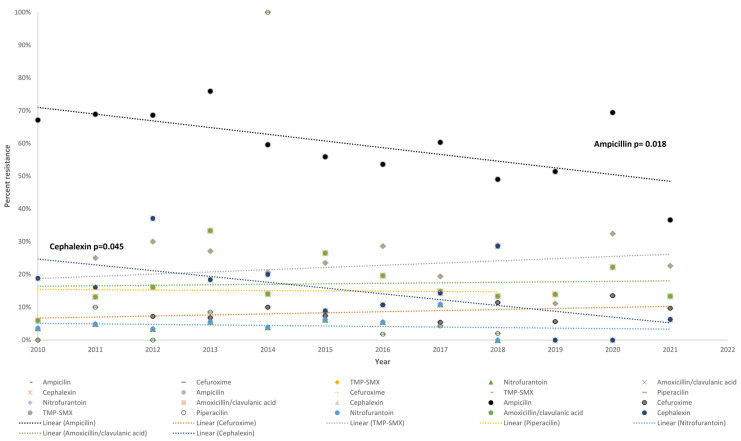
Trends in resistance patterns among all Gram-negative uropathogens for selected antibiotics: ampicillin, cefuroxime, TMP-SMX, nitrofurantoin, amoxicillin–clavulanic acid, and cephalexin.

There was a significant decreasing linear trend in resistance for ampicillin (slope = −0.0205, se = 0.007, *p* = 0.018) and cephalexin (b = −0.0177, se = 0.0008, *p* = 0.045) between 2010 and 2021. There were no significant trends in resistance over time for TMP-SMX (b = 0.0068, se = 0.009, *p* = 0.46), piperacillin (b = −0.0104, se = 0.0242, *p* = 0.67), amoxicillin–clavulanic acid (b = 0.0002, se = 0.0036, *p* = 0.82), ceftriaxone (b = 0.0033, se = 0.0029, *p* = 0.29), cefuroxime (b = 0.0023, se = 0.0027, *p* = 0.41), or penicillin (Figure 2). There was no statistically significant difference in trends between the complicated UTI and the non-complicated UTI groups.

## 4. Discussion

The emergence of antibiotic resistance is a one of the biggest global issues, and it has been addressed by international health organizations and previous studies [21,22]. It is estimated to be the leading cause of death worldwide and has the highest healthcare burden in sub-Saharan Africa and South Asia [21].

Uropathogen resistance is reported by several previous studies from numerous regions worldwide [23,24,25,26,27,28,29,30]. Current evidence suggests high ampicillin [23,24,26,27,31], ampicillin–clavulanic acid [24,25], cephalosporine [23,24,26], and trimethoprim–sulfamethoxazole (TMP-SMX) [25,26,30] resistance patterns for *E. coli* and other uropathogens.

In our study, F-UTI in all age groups and in both the non-complicated UTI and complicated UTI groups was predominantly found in female patients, comprising approximately 90% of the cohort. The prevalence among febrile infant girls is twice that of boys [17]. In Israel, most males are circumcised, which is known to reduce the risk of F-UTI. Research suggests that the prevalence of F-UTI is 2% in circumcised males compared with 21% in uncircumcised males [32,33]. In our cohort, most F-UTI in boys presented at a younger age, during the first months of life. Circumcision was performed in the majority of male patients with F-UTI, and more than half of them had urinary tract abnormalities, which was a similar proportion to that observed in the uncircumcised group. However, given the small number of boys in our sample, a larger uncircumcised cohort might reveal more cases, which could further emphasize the close association between male sex and congenital anomalies in febrile UTI; which should always include imaging as part of the workup [34].

In our study, *E. coli* was the most common uropathogen (85.9%), followed by *P. aeruginosa* (3.5%) and *Klebsiella* spp. (3.4%). A lower prevalence was noted for *Proteus* spp. and *Enterococcus* spp. Similarly, Eramenko et al. [25] examined the bacterial distribution among children aged 3 months to 18 years with UTIs and treated as outpatients in a large community clinic in Israel between 2015 and 2017. *E. coli* was the most common causative bacteria (78.1%), followed by *Proteus* spp. (11.2%), *Klebsiella* spp. (3.9%), and *Enterococcus* spp. (3.4%) [25]. The higher incidence of *P. aeruginosa* in our study could be attributed to the inpatient population, who are more likely to have complicated or recurrent infections and may be exposed to different antibiotic treatments and healthcare-associated pathogens compared with the outpatient population. Similarly, *P. aeruginosa* was frequently identified (5.75%) in a Romanian study by Duicu et al. [35] and among an inpatient pediatric population who had risk factors such as recent antibiotic use or anatomical abnormality [36].

*E. coli* was the most prevalent pathogen in both the non-complicated UTI group and the complicated UTI group, which is a similar result to other studies [37]. Additionally, *E. coli* was significantly more common in the non-complicated UTI group (88% vs. 75%). It’s prevalence was lower in the complicated UTI group, which had a higher incidence of *P. aeruginosa,* reflecting the change in prevalence and the higher pathogen diversity in children at risk for UTIs [36,38]. Laboratory markers like CRP, urea, and creatinine were similar across both cohorts, consistent with their role as indicators of infection severity and renal involvement rather than recurrence-specific changes.

Herein, we report high resistance rates to ampicillin, TMP-SMX, amoxicillin–clavulanic acid, and cephalexin. These resistance rates show both alignment and some discrepancies when compared with other studies. Cag et al. reported a high ampicillin resistance rate of 66.6%, which aligns with our findings of 60.9% resistance [23]. Silva et al. reported a 20.3% resistance for amoxicillin–clavulanic acid and a 14.9% resistance for cephalexin, similar to our findings of 17.3% and 17.8%, respectively [39]. Gunduz and Uludağ Altun reported TMP-SMX, ceftriaxone, and ciprofloxacin resistance rates of 29.8%, 2.7%, and 7.5%, which are comparable to our findings of 24.5%, 4.4%, and 6.6%, respectively [26].

However, there were some discrepancies. For instance, Gunduz and Uludağ Altun reported a higher resistance rate for cefuroxime (28.7%) than what we did (8.6%) [26]. Similarly, Kalaitzidou et al. reported an amoxicillin–clavulanic acid resistance of 28.6%, which is higher than our finding of 17.3% resistance [27]. Lastly, Swerkersson et al. reported nitrofurantoin resistance of 1%, which is lower than our rate of 5.1% [30]. These variations could be attributed to differences in regional prescribing practices, sample sizes, and population demographics. While our findings are largely consistent with those reported in the literature, the discrepancies highlight the need for ongoing surveillance and tailored antibiotic stewardship programs to address regional variations in resistance patterns.

Resistance rates for TMP-SMX, cephalexin, amoxicillin–clavulanic acid, cefuroxime, and ceftriaxone were higher in complicated UTI cases compared with non-complicated UTI cases. Pyelonephritis is treated with a long course (10–14 days) of antibiotics. Children that are receiving repeat courses and children that are on prophylaxis are at risk for developing resistant pathogens. The most prevalent antibiotic for oral treatment following hospital admission is a first-generation cephalosporin. *E. coli* shows increasing resistance to amoxicillin, cephalexin, and even to amoxicillin–clavulanic acid, particularly in the pediatric community where these antibiotics are extensively used for treating conditions like pneumonia, otitis media, and tonsillitis. Previous antibiotic treatment is a significant risk factor for encountering resistant organisms in F-UTI [40].

We also observed a decrease in ampicillin resistance from 2010 to 2021, potentially due to adherence to the 2014 Ministry of Health guidelines, which emphasize using urine culture, reducing antimicrobial prophylaxis, limiting parenteral antimicrobials, and encouraging early switching from empirical to targeted treatment [41]. Updated local guidelines for treating pneumonia, otitis media, and tonsillitis with a focus on antibiotic stewardship likely contributed to this improvement. Reassessing pathogen resistance patterns following these guidelines suggests that a third-generation cephalosporin is a reasonable choice for an inpatient’s first F-UTI, as noted by Kantamalee et al. [7] and other therapy recommendations [42,43]. Moreover, third-generation cephalosporins are particularly advantageous in the pediatric population due to their lower nephrotoxicity compared with gentamicin [44,45]. This reduced risk of kidney damage makes third-generation cephalosporins a safer option for treating children with F-UTI, especially when considering the need to balance efficacy with safety in this vulnerable population.

Children with recurrent F-UTI exhibited significantly higher resistance rates to cephalexin and TMP-SMX compared with those that had non-complicated UTI. No significant differences were found for other antibiotics, including ceftriaxone, used for empirical inpatient treatment. Previous studies [16] have identified recurrent UTI and the continuous use of antibiotic prophylaxis, particularly with cephalosporins, as key predisposing factors that contribute to the development of antibiotic-resistant F-UTI. In our study, the small number of patients on prophylaxis limited further analysis of this subgroup.

During hospitalization, the average time frame for treating F-UTI was 3 days. Patients were discharged when they displayed a significant decrease in inflammatory markers and when their fever resolved. The trend of a short intravenous treatment of 2–4 days and an oral course of 5 days has been studied recently [46,47,48], where it was found to be both safe and beneficial in minimizing antibiotic use and reducing the risk of encountering resistance in future infections.

In a meta-analysis by Bryce et al., which evaluated 77,783 *E. coli* isolates from 58 observational studies, resistance rates were compared between Organization for Economic Co-operation and Development (OECD) countries and non-OECD countries. Resistance rates were reported for ampicillin (53.4% vs. 79.8%), amoxicillin–clavulanic acid (8.2% vs. 60.3%), ciprofloxacin (2.1% vs. 26.8%), and nitrofurantoin (1.3% vs. 17.0%) [49]. Overall, while our results show higher resistance rates than those in OECD countries, they are lower compared with non-OECD countries. This pattern may indicate that while our setting faces more significant resistance challenges than OECD countries, it does not experience the extreme levels of resistance seen in non-OECD countries. Such a middle ground might reflect regional differences in antibiotic use practices, healthcare infrastructure, and infection control measures.

This study’s generalizability is limited due to certain factors, including its retrospective design in a single center and the exclusion of outpatient children with UTIs. Therefore, caution is necessary when applying the results for nationwide or other population purposes. Additionally, the study did not analyze functional abnormalities of the lower urinary tract. Although they are not very common in the pediatric population, they could impact the findings. Excluding infants under 3 months old from the study may limit our understanding of UTI and uropathogen resistance in this vulnerable population. Furthermore, the isolated uropathogens were not preserved for subsequent analysis of antibiotic resistance genes. The small sample size limits our ability to draw definitive conclusions about the effect of circumcision. However, the number of urinary tract anomalies identified in both circumcised and uncircumcised boys is noteworthy and underscores the importance of imaging in complicated UTI cases. Lastly, our study is particularly noteworthy regarding the sex distribution of patients as it predominantly included female patients. This is significant given that most male children in Israel are circumcised, a factor that likely impacts the prevalence of UTIs. The fact that most children in our study were female underscores the importance of circumcision in reducing UTI incidence in males.

## 5. Conclusions

*E. coli* was the predominant uropathogen, followed by other Gram-negative bacteria. High resistance rates were observed for ampicillin, TMP-SMX, amoxicillin–clavulanic acid, and cephalexin, highlighting the need to consider these patterns when selecting empirical outpatient treatments. Complicated UTI cases showed increased prevalence of *Pseudomonas aeruginosa* and *Klebsiella pneumoniae*, with higher cephalexin resistance, emphasizing the importance of tailored treatment. Low resistance to ceftriaxone and gentamicin suggests that ceftriaxone, with a lower nephrotoxicity, is a suitable parenteral antimicrobial option for treating severe community-acquired F-UTI.

## Figures and Tables

**Table 1:** Clinical characteristics of inpatient children diagnosed with F-UTI (N = 761).

Characteristic	Complicated UTI (N = 453)	Non-Complicated UTI (N = 308)	*p*
Male sex	59 (13.0)	0 (0.0)	---
Median patient age (mo)	17.5 [9.0–48.25]	21 [9–54.5]	0.18
Urine tract pathology	266/443 * (60.0%)	N/A	
Hydronephrosis	191 (43.1)		
Duplex collecting system	14 (3.1)		
Vesico-ureteric reflux	11 (2.5)		
Stenosis	2 (0.5)		
Other	48 (10.8)		
CRP (mg/dL)	86.75 [39.75–153.25]	84.50 [34.00–155.00]	0.46
Max CRP (mg/dL)	103.50 [43.75–170.00]	95.30 [47.78–169.80]	0.32
Urea (mg/dL)	20.0 [16–25]	20.0 [16–25]	0.93
Creatinine (mg/dL)	0.30 [0.30–0.40]	0.30 [0.30–0.50]	0.52
Pathogen			
*Escherichia coli*	368 (81.2)	286 (92.9)	<0.001
*Klebsiella pneumoniae*	21 (4.6)	5 (1.6)	0.03
*Pseudomonas aeruginosa*	24 (5.3)	3 (1.0)	0.002
*Proteus mirabilis*	14 (3.1)	7 (2.3)	0.65
*Enterococcus faecalis*	12 (2.6)	3 (1.0)	0.10
*Enterobacter* spp.	5 (1.1)	1 (0.3)	0.41
*Staphylococcus aureus*	5 (1.1)	1 (0.3)	0.41
*Citrobacter* spp.	1 (0.2)	2 (0.6)	0.57
Other	3 (0.7)	1 (0.3)	0.65

Data are expressed as numbers and (percents) or medians [interquartile ranges]. Chi-square or Fisher’s exact tests were performed to compare categorical variables between groups, and the Mann–Whitney test was performed to compare continuous variables with skewed distribution. * Ten patients did not undergo urinary tract sonography. Abbreviations: UTI, urinary tract infection; CRP, C-reactive protein; IQR, interquartile range. A *p* value ≤ 0.05 was considered significant.

**Table 2:** Etiology of UTI by age group.

	All (N = 761)	3–11 mo (N = 230)	1–2 yr (N = 192)	3–4 yr (N = 136)	5–11 yr (N = 131)	12–17 yr (N = 72)	*p*
*Escherichia coli*	654 (85.9)	201 (87.4)	171 (89.1)	106 (77.9)	113 (86.3)	63 (87.5)	0.072
*Klebsiella pneumoniae*	26 (3.4)	9 (3.5)	5 (2.6)	5 (3.7)	5 (3.8)	2 (2.8)	0.96
*Pseudomonas aeruginosa*	27 (3.5)	5 (2.2)	7 (3.6)	10 (7.4)	2 (1.5)	3 (4.2)	0.086
*Proteus mirabilis*	21 (2.8)	3 (1.3)	5 (2.6)	9 (6.6)	4 (3.1)	0 (0.0)	0.033
*Enterococcus fecalis*	15 (2.0)	5(2.2)	3 (1.6)	3 (2.2)	3 (2.3)	1 (1.4)	0.98
*Enterobacter* spp.	6 (0.8)	2 (0.9)	1 (0.5)	1 (0.7)	1 (0.8)	1 (1.4)	0.94
*Staphylococcus aureus*	6 (0.8)	0 (0.0)	0 (0.0)	1 (0.7)	3 (2.3)	2 (2.8)	0.008
*Citrobacter* spp.	2(0.3)	2 (0.7)	0 (0.0)	0(0.0)	0 (0.0)	0 (0.0)	0.73
Other	4 (0.6)	3 (1.3)	0 (0.0)	0 (0.0)	0 (0.0)	1 (1.4)	0.16

Data are expressed as numbers and (percents). Chi-square or Fisher’s exact tests were performed to compare categorical variables between groups. A *p* value ≤ 0.05 was considered significant.

**Table 3:** Antimicrobial resistance for pediatric patients admitted with F-UTI.

	All Patients with Cultured Gram-Negative Bacteria (N = 739) Resistant/Number Tested (%)	Complicated UTI Resistant/Number Tested (%)	Non-Complicated UTI Resistant/Number Tested (%)	*p* Value
Ampicillin	352/579 (60.9)	216/347 (62.3)	136/231 (58.3)	0.42
TMP-SMX	107/436 (24.5)	71/275 (25.8)	36/161 (22.4)	0.42
Cephalexin	76/426 (17.8)	41/212 (19.3)	35/213 (16.4)	0.03
Amoxicillin–clavulanic	104/600 (17.3)	58/346 (16.8)	46/243 (18.2)	0.50
Cefuroxime	34/397 (8.6)	24/237 (10.1)	10/160 (6.2)	0.18
Ciprofloxacin	31/472 (6.6)	21/307 (6.8)	10/165 (6.1)	0.75
Nitrofurantion	26/472 (5.2)	17/267 (6.4)	9/236 (3.8)	0.20
Ceftriaxone	26/597 (4.4)	17/344 (4.9)	9/252 (3.6)	0.42
Gentamicin	38/632 (6.0)	25/375 (6.7)	13/256 (5.1)	0.41

Data are expressed as numbers and (percents). Chi-squared tests were performed to compare categorical variables between groups. A *p* value ≤ 0.05 was considered significant.

## Data Availability

The data presented in this study are available on request from the corresponding author.
